# E-Cigarette- and Vaping-Related Lung Injury (EVALI) at a Regional Hospital System in South Carolina

**DOI:** 10.1155/2020/5370606

**Published:** 2020-05-17

**Authors:** Daniel Temas, Armin Meyer

**Affiliations:** University of South Carolina School of Medicine-Greenville/Prisma Health-Upstate, 701 Grove Rd., Greenville, SC 29605, USA

## Abstract

We report on four cases of severe lung injury and respiratory failure attributed to E-cigarette use that presented between July and August, 2019. The patients described were relatively healthy without clinically significant history of lung disease. Each developed severe acute respiratory distress shortly following E-cigarette use. In each case, the patients initially presented with considerable hypoxia and infectious-appearing pattern with elevated inflammatory markers on laboratory values. Imaging studies demonstrated a consistent pattern of widespread bilateral interstitial infiltrates with a medial distribution. All but one of the cases involved the admitted use of THC oil in E-cigarettes. There was rapid progression of illness requiring increased supplemental oxygen and in two cases, requiring urgent intubation and mechanical ventilation. No infectious organism was isolated in any case, and patients improved rapidly with the initiation of steroids. These are among the first cases reported in South Carolina and are consistent with similar cases that have been reported around the country.

## 1. Introduction

E-cigarette use (“vaping”) has exploded in popularity over the last 10 years. Now, approximately 2.8% of Americans are current E-cigarette users per CDC statistics [1]. Growth in use is particularly marked in youth demographics with 4.9% of middle school students and 20.8% of high school students reporting vaping within 30 days of the survey [1]. In spite of its rapid market growth, exploration of the short- and long-term health effects of E-cigarettes has been limited. However, initial data compiled by Klomparens et al. suggests an association between E-cigarette use and lung disease, as well as increased risk of cardiovascular disease [2]. Furthermore, due to the variations in manufacturing of these products and lack of regulatory oversight, there are wide variations in quality and composition of the products offered.

E-cigarettes are composed of a battery-powered heating element that heats a liquid substance (“e-juice”) which generally contains propylene glycol, glycerine, flavoring, and nicotine in various concentrations, in addition to various other chemical components. This liquid is absorbed into a cotton wick. The cotton wick is then heated, and the absorbed liquids form an aerosol inhaled into the lungs.

In recent years, vaping of cannabis oil or other preparations of THC have increased in popularity as an alternative to traditional methods of consuming cannabis [3]. Many of these preparations are manufactured without uniform standards or quality control and sold illicitly or online from unverifiable sources. There has been at least one case report describing acute lung injury due to vaporized cannabis oil [4]. Several of the chemical components, including flavoring agents, have been identified as potentially toxic [5]. Vitamin E (alpha-tocopherol) acetate in particular has been implicated as a potential causative agent, based on BAL samples from affected patients [6].

This increase in the popularity of E-cigarette use has coincided with an increased number of reported cases of severe lung disease associated with or directly attributed to vaping. The first officially reported death attributed to E-cigarette use in America was reported in Illinois in August 2019 [7]. Subsequently, multiple additional cases of severe illness and mortality attributed to vaping have been reported around the country. This prompted the CDC to develop a case definition for E-cigarette-associated lung injury which includes the following criteria [8]:

- E-cigarette use within 90 days
- With pulmonary infiltrates and ground-glass opacities on imaging
- Negative respiratory viral panel and influenza screen and absence of any other identifiable disease-causing pathogen

Here, we present four cases of E-cigarette- and vaping-related lung injury (EVALI) in a regional health system in South Carolina treated between July and August 2019. Initial presentation, evaluation, and hospital treatment course are described.

## 2. Cases

### 2.1. Case 1

A 43-year-old female patient presented to the Emergency Department via EMS following 3-4 days of reported cough, confusion, and fever up to 38.9°C. Her prior medical history was significant for 1-pack-per-day cigarette smoking and polysubstance abuse. On EMS arrival, she was noted to have an O_2_ saturation of 50% on room air. On physical exam at presentation, she was noted to have dry rales throughout bilateral lung fields. The patient was in possession of a vape pen, along with paraphernalia including a “Dank Vapes” THC oil cartridge labeled “92.33% THC.” The patient denied any environmental exposures or significant sick contacts. She was emergently intubated in the Emergency Department due to hypoxia and altered mental status.

**Figure 1:**
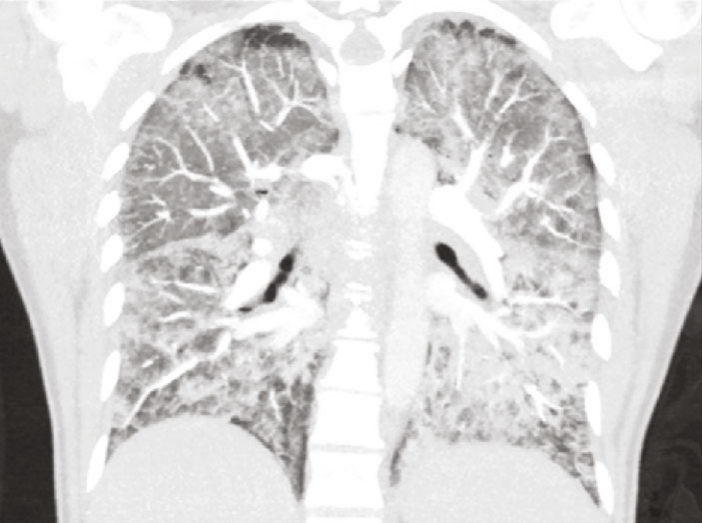
CT angiogram of the chest demonstrating widespread bilateral ground-glass opacities with some basilar and dependent predominance.

Initial lab studies were significant for white blood cell (WBC) of 11,100/mm^3^ with 17% bands, lactate 5.35 mmol/L, C-reactive protein (CRP) 315.7 mg/L, and procalcitonin 5.14 ng/mL. Urine drug screen resulted positive for cannabinoids and benzodiazepines. B-natriuretic peptide (BNP) on admission was 326 pg/mL, which rose to 1195 pg/mL on the second day of hospitalization. Hemoglobin dropped from 11.4 g/dL to 8.4 g/dL on hospital day 3 without obvious evidence of bleeding. Arterial blood gas on 100% FiO_2_ demonstrated pH 7.38, pCO_2_ 42 mmHg, and pO_2_ 65 mmHg. CT angiogram of the chest (Figure 1) demonstrated diffuse bilateral ground-glass opacities with some dependent and basilar predominance and no acute focal consolidation. Respiratory culture resulted with light growth yeast. Respiratory pathogen viral PCR (RPP) was negative for acute viral illness. Blood and urine cultures collected on admission were negative. HIV screen was nonreactive. Bronchoscopy demonstrated normal appearance of bilateral bronchioles to segmental levels with minimal secretions throughout. Bronchoalveolar lavage (BAL) cultures were negative. BAL cytology demonstrated WBC of 1195/mm^3^ with neutrophilic predominance and 2900/mm^3^ RBC. Pathology further noted presence of mixed leukocytes and alveolar macrophages.

The patient was initially treated empirically for sepsis with broad-spectrum antibiotics. She continued to require 100% FiO_2_ on hospital day 2, and intravenous methylprednisolone 500 mg daily was initiated. Her respiratory status subsequently improved, and she was extubated on hospital day 3 to heated high-flow nasal cannula, initially at 60% FiO_2_. Steroid treatment was transitioned to oral prednisone 60 mg twice daily. Oxygen requirement was progressively weaned to 6 L via nasal cannula over the subsequent 48 hours. Patient would ultimately leave against medical advice on hospital day 5 while still requiring 4-6 L supplemental oxygen.

### 2.2. Case 2

A 33-year-old male patient presented with a 2-day history of cough, shortness of breath, and fever. Medical history was significant for remote history of asthma as a child and hospitalization for uncomplicated community-acquired pneumonia 2 years prior to presentation. He was not taking any chronic medications. He reported smoking approximately 1 pack of cigarettes daily and regular e-cigarette use. He endorsed he had increased smoking due to stress over the preceding week and had vaped “all night” prior to presentation. He denied THC use.

Initial vital signs were significant for hypoxia, tachycardia, and temp of 38.0°C. There were decreased breath sounds bilaterally but no wheezes, rales, or rhonchi noted on physical exam. Patient was started on supplemental oxygen via nasal cannula at 4-5 L.

**Figure 2:**
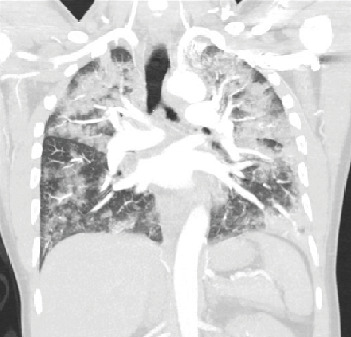
CT angiogram of the chest demonstrating diffuse multifocal ground-glass opacities with peripheral sparing and left pleural effusion.

Initial labs were significant for WBC of 18,100/mm^3^ and procalcitonin of 0.08 ng/mL. The patient's hemoglobin fell from 15.4 g/dL on initial labs to 12.1 on hospital day 3. Chest X-ray demonstrated bilateral infiltrates. CT angiogram of the chest demonstrated diffuse, multifocal bilateral ground-glass opacities with relative peripheral sparing. Reactive mediastinal/hilar lymphadenopathy along with bilateral pleural effusions was also noted (Figure 2). Urine toxicology screen was not performed. RPP was negative, as were blood and respiratory cultures. HIV screen was also negative. Bronchoscopy was not performed in this patient.

Patient was initially treated empirically with ceftriaxone and azithromycin. He became more dyspneic and hypoxic over the 24 hours following admission requiring increase in supplemental oxygen to high-flow nasal cannula at 8 L. On hospital day 3, he was started on methylprednisolone 125 mg IV daily. Clinical condition subsequently improved and supplemental O_2_ requirement was weaned to room air on day of discharge (hospital day 6). He was discharged with a 4-week prednisone taper, starting with 60 mg daily, and PRN albuterol inhaler.

### 2.3. Case 3

A 20-year-old male presented to the ED with epigastric pain and intractable nausea and vomiting. He also reported 3 days of nonproductive cough, shortness of breath, and fever for three days. There were no reported sick contacts or toxic exposures. Patient was a never-smoker and denied drug use but admitted to E-cigarette use.

On presentation, the patient was normotensive and tachycardic with a rate of 120 bpm, with O_2_ saturation of 94% on room air. Physical exam was only remarkable for mild abdominal pain. Labs were significant for WBC of 15,800/mm^3^, procalcitonin 0.86 ng/mL, and lactate 3.33 mmol/L. Hemoglobin dropped from 15.4 g/dL on presentation to 10.6 g/dL on hospital day 7 without evidence of frank bleeding. Urine drug screen was positive for cannabinoids. Blood cultures, respiratory cultures, and RPP were negative. HIV was also negative.

**Figure 3:**
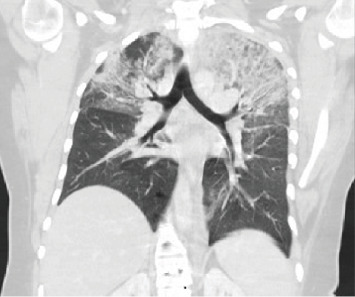
CT of the chest without contrast demonstrating bilateral upper lobe ground-glass opacities.

On hospital day 3, the patient became acutely febrile with temperature up to 39.1°C, tachycardic, short of breath, and hypoxic requiring up to 6 L O_2_ via nasal cannula. CT of the chest (Figure 3) demonstrated bilateral upper lobe-predominant consolidations with overlying ground-glass opacities. Follow-up labs demonstrated an erythrocyte sedimentation rate (ESR) of 51 mm/hr and CRP > 320.0 mg/L. Arterial blood gas demonstrated pH 7.45, pCO_2_ 33 mmHg, and pO_2_ 52 mmHg. His supplemental O_2_ was increased to heated high-flow nasal cannula at 70% FiO_2_. He was subsequently started on steroid treatment with methylprednisolone 1000 mg IV daily.

Oxygen requirement was successfully weaned over the next several days. At time of discharge, the patient was maintaining adequate O_2_ saturations on room air at rest but continued to require 2 L via nasal cannula with ambulation. Patient was discharged on hospital day 8 with a 6-week steroid taper, starting with Prednisone 60 mg PO daily.

On chart review follow-up, patient reported ongoing dyspnea on exertion and shortness of breath limiting daily activities 6 months following initial presentation and hospitalization. Further workup and testing are in the process at the time of this report.

### 2.4. Case 4

A 19-year-old male presented to the ED with 3 days of headache, malaise, abdominal pain, and nausea/vomiting. He also reported shortness of breath and cough with occasional scant hemoptysis. He had no significant prior medical history and took no medications. He denied any significant sick contacts or exposures. Patient was reportedly a nonsmoker but admitted to vaping marijuana.

Vital signs are significant for tachycardia at a rate of 103 bpm and temp of 38.5°C. Oxygen saturation was 94% on 4-liter supplemental oxygen. Bibasilar rales were noted on auscultation. The initial labs were significant for WBC of 12,400/mm^3^, procalcitonin 0.27 ng/mL, CRP > 320.0 mg/L, and ESR 100 mm/hr. Initial chest X-ray demonstrated bibasilar consolidations without definite focal infiltrate. Urine drug screen was positive for cannabinoids. Hemoglobin fell from 14.6 g/dL on the day prior to presentation to 11.0 g/dL on hospital day 3. Respiratory cultures and RPP were negative. Due to the severity of headache, a lumbar puncture was performed. Opening pressure within normal limits at 26 cm H_2_O and spinal fluid analysis was unremarkable.

**Figure 4:**
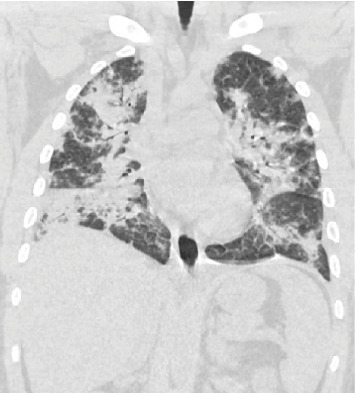
CT of the chest without contrast demonstrating patchy bilateral ground-glass opacities and small bilateral pleural effusions.

Patient was started on empiric antibiotics for suspected community-acquired pneumonia. He became progressively more hypoxemic over the next 4 days requiring escalation from 4 L O_2_ via nasal cannula to high flow. He underwent bronchoscopy which demonstrated airways without noted structural abnormalities to segmental branches bilaterally and minimal secretions. BAL cytology demonstrated neutrophilic predominance. Pathology review noted acute inflammatory background with blood and numerous macrophages; occasional clusters of abnormal cells were also noted. Follow-up CT of the chest (Figure 4) demonstrated dense, patchy bilateral ground-glass opacities, bilateral lower lobe consolidations with air bronchograms, and small bilateral pleural effusions.

On hospital day 4, the patient became acutely hypoxic with increasing oxygen requirement. He was intubated for suspected Acute Respiratory Distress Syndrome (ARDS), requiring 100% FiO_2_ to maintain adequate oxygen saturations. He was subsequently started on steroid therapy with methylprednisolone 250 mg IV twice daily. Respiratory status gradually improved on mechanical ventilation, and steroid dose was weaned to methylprednisolone 60 mg IV every 8 hours on day 3 of mechanical ventilation. The patient was extubated after a total of 4 days of mechanical ventilation. He was transitioned to prednisone 60 mg PO daily. Oxygen requirement and overall clinical status gradually improved over the next several days, and the patient was tolerating room air at time of discharge on hospital day 10. He was discharged on a prolonged steroid taper starting with prednisone 60 mg PO daily.

## 3. Discussion

In the cases described above, the patients presented with acute hypoxia, respiratory distress, and diffuse inflammatory pattern on imaging. Patient age ranged from 19 years old to 43 years old, three male and one female. All of the described patients admitted to E-cigarette use; all but one admitted to vaping THC. Initial presentation in all cases was concerning for infection with fever and leukocytosis, but in all cases, no organism was identified. The patients described were without significant prior pulmonary disease. In each case, the patients were found to have severe hypoxemia requiring significant supplemental oxygen to maintain adequate oxygenation. In two of the four cases, intubation and mechanical ventilation were required due to the severity of hypoxemia. All received steroids at varying doses with subsequent improvement in symptoms and hypoxia. Each of these cases fit the CDC case definition for EVALI [8] as described above.

Some other commonalities among these cases include modest elevation in WBC and significantly elevated CRP (Table 1). Procalcitonin was notably elevated in case 1 but the remaining cases had only modest elevations. Imaging studies in all cases demonstrated bilateral pulmonary opacities without focal consolidation. The elevated CRP and clinical response to steroids suggest a reactive inflammatory process. E-cigarette use has previously been described to cause inflammatory responses similar to cigarette smoking [2, 9]. Further investigation will be required to determine the correlation between inflammatory markers and E-cigarette use; however, measurement of C-reactive protein and other inflammatory markers may be warranted in suspected cases. Additionally, all patients experienced a significant drop in hemoglobin, averaging -3.675 g/dL drop in hemoglobin without evidence of bleeding. The significance of this is unclear; however, this could also be related to the acute inflammatory state suggested by the elevated CRP.

## 4. Limitations

There were several notable limitations to our evaluation. The evaluation and treatment of the patients described above occurred between July and August, 2019. This was early in the timeline of reported cases of EVALI and was not a commonly recognized clinical syndrome. As such, standards of care for evaluation and treatment of potential cases were not established. This accounts for lack of bronchoscopic evaluation in cases 2 and 3, as well as the varied steroid dosing regimens. Additionally, case 2 did not have a measured CRP level and, while there was a high clinical suspicion, there was no urine toxicology screen to confirm THC use. Furthermore, these cases were identified prior to the NEJM article published in December 2019 identifying vitamin E acetate as a potential causative agent [6]; as such, it was not measured on the BAL samples.

## 5. Conclusion

The cases described represent only a small sample of an alarming trend across the country. A growing number of cases are being described of severe and life-threatening pulmonary illness with E-cigarette use and more specifically THC-based products, as a potential culprit. This is further supported by the data surrounding the presence of vitamin E acetate in these products and its suspected role in contributing to EVALI [10]. Furthermore, of 2022 cases of EVALI identified by the CDC, 82% reported the use of THC-containing products [10].

With this growing incidence, appropriate and prompt identification of these cases and initiation of appropriate treatment will be essential to minimizing associated mortality. The infectious-appearing presentation and imaging findings as described above, along with the appropriate history, should raise clinical suspicion for this disease process. As we have reported, prompt initiation of high-dose corticosteroid treatment is associated with improvement in oxygenation and overall clinical condition.

While some initial strides have been made to identify the underlying etiology, further research will be essential to both identifying the agent (s) responsible for the lung injury and establishing standardized evaluation and treatment protocols. Furthermore, it remains to be seen what the long-term consequences of this clinical syndrome present for the affected patients. Outpatient follow-up and appropriate clinical monitoring with serologies, imaging, and pulmonary function testing would be warranted.

At the time of completion of this report, at least 10 additional cases of vaping-associated lung injury have been identified within the hospital system.

## Figures and Tables

**Table 1:** Lab value similarities among observed cases.

Case	Leukocyte count (TH/mm^3^)	Procalcitonin (ng/mL)	CRP (mg/L)	Change in hemoglobin (g/dL)
1	11,000	5.14	315.7	-3.0
2	18,100	0.08	~	-3.3
3	15,800	0.86	>320	-4.8
4	12,400	0.15	>320	-3.6

## Data Availability

Not required.
